# *Porphyromonas gingivalis* suppresses invasion of *Fusobacterium nucleatum* into gingival epithelial cells

**DOI:** 10.1080/20002297.2017.1320193

**Published:** 2017-06-12

**Authors:** Young-Jung Jung, Hye-Kyoung Jun, Bong-Kyu Choi

**Affiliations:** ^a^ Department of Oral Microbiology and Immunology, School of Dentistry, University of Louisville, KY, USA; ^b^ Dental Research Institute;Seoul National University, Seoul, Republic of Korea

**Keywords:** Gingipain, mixed infection, periodontopathogens, gingival epithelial cells, invasion, PI3K/AKT pathway

## Abstract

Invasion of periodontal pathogens into periodontal tissues is an important step that can cause tissue destruction in periodontal diseases. *Porphyromonas gingivalis* is a keystone pathogen and its gingipains are key virulence factors. *Fusobacterium nucleatum* is a bridge organism that mediates coadhesion of disease-causing late colonizers such as *P. gingivalis* and early colonizers during the development of dental biofilms. The aim of this study was to investigate how *P. gingivalis*, in particular its gingipains, influences the invasion of coinfecting *F. nucleatum* into gingival epithelial cells. When invasion of *F. nucleatum* was analyzed after 4 h of infection, invasion of *F. nucleatum* was suppressed in the presence of *P. gingivalis* compared with during monoinfection. However, coinfection with a gingipain-null mutant of *P. gingivalis* did not affect invasion of *F. nucleatum*. Inhibition of PI3K reduced invasion of *F. nucleatum*. *P. gingivalis* inactivated the PI3K/AKT pathway, which was also dependent on gingipains. Survival of intracellular *F. nucleatum* was promoted by *P. gingivalis* with Arg gingipain mutation. The results suggest that *P. gingivalis*, in particular its gingipains, can affect the invasion of coinfecting *F. nucleatum* through modulating intracellular signaling of the host cells.

## Introduction

Chronic periodontitis is not only one of the leading causes of tooth loss, but it is also an important risk factor for multiple systemic diseases, such as rheumatoid arthritis and cardiovascular disease [1]. Polymicrobial biofilms present in subgingival crevices are the most important etiological factor in the pathogenesis of periodontitis. Polymicrobial communities develop through interspecies interactions and adaptation within the surrounding microenvironments, and more than 400 species of bacteria have been detected in subgingival biofilms [2,3].

*Porphyromonas gingivalis*, a Gram-negative obligatory anaerobe, has been classified into a disease-causing ‘red complex’ [4]. Recently it was demonstrated that *P. gingivalis* acts as a keystone pathogen in the pathogenesis of periodontitis [5]. Subversion of host immune surveillance by *P. gingivalis* creates an environment that facilitates dysbiosis of subgingival microbiota, and the dysbiotic microbiota with increased pathogenicity overactivate inflammation in periodontal tissues [5]. Gingipains (Arg-X-specific Arg gingipains [RgpA and RgpB] and Lys-X-specific Lys gingipain [Kgp]), major proteases of *P. gingivalis*, are essential for its pathogenic potential [6–8] and are often considered to be potential therapeutic targets. Along with other virulence factors, such as lipopolysaccharide, fimbriae, and several other proteases, gingipains contribute to the ability of *P. gingivalis* to dysregulate host immune systems [9,10].

*Fusobacterium nucleatum* is a spindle-shaped, Gram-negative anaerobe frequently and abundantly found in the oral cavity under both healthy and diseased conditions [11]. The importance of *F. nucleatum* in the development of polymicrobial biofilms has long been recognized. *F*. *nucleatum* binds to early colonizers and acts as a bridging organism that mediates coadherence of disease-causing late colonizers, including *P. gingivalis*, to dental biofilms [12]. Several adhesins, including FomA, aid1, Fap2, and RadD, are involved in interactions with various oral bacteria, such as oral streptococci and *P. gingivalis* [13–15]. Other virulence factors such as GroEL and FadA contribute to the ability of *F. nucleatum* to cause several systemic diseases, such as atherosclerosis and colorectal cancer [16,17].

Invasion of periodontal pathogens into periodontal tissues is an important step that can initiate periodontal diseases [18]. Gingival epithelium is the first cell layer that bacteria in subgingival biofilms have to pass through in order to invade into the deeper parts of periodontal tissues. *P*. *gingivalis* invades into gingival epithelial cells via interactions of its fimbriae with β1 integrins of the cells [19], while invasion of *F. nucleatum* into gingival epithelial cells is mediated by its FadA [20]. *F*. *nucleatum* has been shown to enhance the invasion of *P. gingivalis* [21,22]. However, the effect of *P. gingivalis* on *F. nucleatum* invasion has not yet been studied. This study reports that *P. gingivalis* coinfection decreases *F. nucleatum* invasion into gingival epithelial cells in a gingipain-dependent manner, possibly via modulating the PI3K/AKT pathway.

## Methods

### Bacteria and cell culture

*F*. *nucleatum* ATCC 25586 was grown in brain heart infusion (BHI) broth supplemented with 5 μg/mL of hemin and 1 μg/mL of vitamin K under anaerobic conditions (10% H_2_, 10% CO_2_, and 80% N_2_) at 37°C. Gingipain mutants of *P. gingivalis* ATCC 33277, KDP129 (*kgp*^–^), KDP133 (*rgpA*^–^ *rgpB*^–^), and KDP136 (*kgp*^–^ *rgpA*^–^ *rgpB*^–^) were kindly provided by Dr Koji Nakayama of Nagasaki University, Nagasaki, Japan. Wild-type *P. gingivalis* (ATCC 33277) and the mutant strains were grown anaerobically at 37°C in enriched BHI broth supplemented with 5 μg/mL of hemin and 1 μg/mL of vitamin K, and the following antibiotics, as described previously [6]: chloramphenicol (20 μg/mL), erythromycin (10 μg/mL), and tetracycline (0.7 μg/mL).

Human Oral Keratinocyte-16B (HOK-16B) cells were kindly provided by Dr NH Park, University of California, Los Angeles, CA, and were immortalized by transfecting keratinized gingival epithelium from excised retromolar gingival tissues with human papilloma virus type 16 [23]. The cells were cultured in keratinocyte growth medium containing a supplementary growth factor bullet kit (KGM; Clonetics, San Diego, CA) in a humidified 5% CO_2_ atmosphere at 37°C.

### Intracellular invasion by *F. nucleatum*

HOK-16B cells (1 × 10^5^ cells/well in 24-well plates) were infected with carboxyfluorescein diacetate succinimidyl ester (CFSE; Molecular Probes, Eugene, OR)–labeled *F. nucleatum* in the presence or absence of unlabeled *P. gingivalis* at the indicated multiplicities of infection (MOIs). Invasion by *F. nucleatum* into the cells was analyzed after 4 h of infection because a previous study [24] reported that the percentage of cells containing *F. nucleatum* decreased slightly after 5 h of infection. In the preliminary experiments of *F. nucleatum* monoinfection, the bacterial invasion into the cells increased in a MOI-dependent manner, and a significant increase was observed at a MOI of 500 (data not shown). Accordingly, *F. nucleatum* at a MOI of 500 was used for coinfection with *P. gingivalis*. To determine the role of gingipains in the effect of coinfecting *P. gingivalis* on *F. nucleatum* invasion, wild-type and gingipain mutant strains of *P. gingivalis* were used. To examine the effect of inhibition of phosphatidylinositol-4,5-bisphosphate 3-kinase (PI3K), the cells were preincubated with the indicated concentration of wortmannin (Sigma–Aldrich, St. Louis, MO) for 30 min and then infected with CFSE-labeled *F. nucleatum* at a MOI of 500 in the presence or absence of *P. gingivalis*.

### Flow cytometry

Invasion into HOK-16B cells by *F. nucleatum* was analyzed by flow cytometry, as described previously [24–26], with a slight modification. After 4 h of infection, the cells were detached with trypsin/ethylenediaminetetraacetic acid, and the extracellular fluorescence from attached but not internalized bacteria was quenched using 400 μg/mL of trypan blue to measure fluorescence signal only from intracellular bacteria. The internalization of *F. nucleatum* was then analyzed via flow cytometry (FACSCalibur; BD Biosciences, San Jose, CA). After HOK-16B cells were gated based on forward scatter and side scatter of uninfected cells, the fluorescence intensity of cells in fluorescence channel FL1-H (for green fluorescence) was determined. At least 10,000 cells of each group were analyzed and, compared with uninfected cells (a negative control), cells with intracellular *F. nucleatum* exhibited increased fluorescence intensity. The percentage of cells with intracellular *F. nucleatum* and the mean fluorescence intensity were determined.

### Confocal microscopy

HOK-16B cells (2 × 10^4^ cells/well in 24-well plates) were infected with CFSE-labeled *F. nucleatum* in the presence or absence of unlabeled *P. gingivalis* at a MOI of 500 for 4 h. The infected cells were washed with phosphate-buffered saline (PBS; pH 7.4) and fixed with 4% paraformaldehyde. After permeabilization, the actin cytoskeleton and nuclei were stained with rhodamine phalloidin (Sigma–Aldrich) and Hoechst 33342 (Molecular Probes), respectively. Invasion of *F. nucleatum* into the cells was examined on a confocal laser scanning microscope (LSM 700; Carl Zeiss, Jena, Germany) and ZEN software (Carl Zeiss) with z-sections (10 z-stack images with 1 µm step size) using 405, 488, and 555 nm lasers for Hoechst 33342, CFSE, and rhodamine, respectively.

### Immunoblot

HOK-16B cells (5 × 10^5^ cells/well in six-well plates) were infected with *P. gingivalis* in the presence or absence of *F. nucleatum* at a MOI of 500 for the indicated time. The cells were washed with cold PBS, harvested, and lysed with radioimmunoprecipitation assay buffer. The lysates were separated by sodium dodecyl sulfate-polyacrylamide gel electrophoresis and transferred to polyvinylidene difluoride membranes (Millipore, Billerica, MA). After the membranes were incubated in blocking buffer (Tris-buffered saline containing 0.1% Tween 20 and 5% non-fat dried milk) for 1 h, the blots were probed with the following primary antibodies: rabbit polyclonal anti-phospho-PI3K p85 (Tyr458)/p55 (Tyr199), rabbit polyclonal anti-PI3K p85, rabbit polyclonal anti-phospho-AKT (Ser473), and rabbit polyclonal anti-AKT from Cell Signaling Technology (Beverly, MA), and mouse monoclonal anti-actin from BD Biosciences. The blots were incubated with horseradish peroxidase–conjugated anti-rabbit immunoglobulin G (IgG) and anti-mouse IgG secondary antibodies (R&D Systems, Minneapolis, MN), and binding of the proteins was visualized using ECL Western blotting substrate (SUPEX; Dyne-Bio, Sungnam, Korea).

### Intracellular survival

HOK-16B cells (1 × 10^5^ cells/well in 24-well plates) were infected with *F. nucleatum* in the presence or absence of *P. gingivalis* at a MOI of 500 for 4 h. The infected cells were washed twice with PBS and incubated in media containing 300 μg/mL of gentamicin and 200 μg/mL of metronidazole for 1 h to kill extracellular bacteria. Then, the cells were washed twice with PBS and further incubated in fresh culture media for 12, 18, 24, and 48 h. At each time point, the cells were lysed with 0.5% saponin in PBS for 10 min at room temperature, and the lysates containing viable *F. nucleatum* were 10-fold serially diluted and plated on a BHI agar plate containing 5 μg/mL of hemin, 1 μg/mL of vitamin K, 5% sheep blood, and 15.7 μg/mL of vancomycin. In preliminary experiments, 15.7 μg/mL of vancomycin inhibited the growth of wild-type and gingipain mutant strains of *P. gingivalis*, but the growth of *F. nucleatum* was not affected. Colonies of *F. nucleatum* and *P. gingivalis* on BHI blood agar plates were clearly distinguishable by their color and morphology, and no colony of *P. gingivalis* was observed on the agar plates containing 15.7 μg/mL of vancomycin. After incubation for 4–5 days under anaerobic conditions, the colonies were counted and expressed as percentages of the number of intracellular *F. nucleatum* in monoinfection.

### Statistical analysis

Experiments were carried out in triplicate and independently repeated at least three times. A standard two-tailed *t*-test was used for statistical analysis, and *p*-values of <0.05 were considered significant.

## Results

### Coinfection with *P. gingivalis* curtails invasion of *F. nucleatum* into gingival epithelial cells

**Figure 1.**
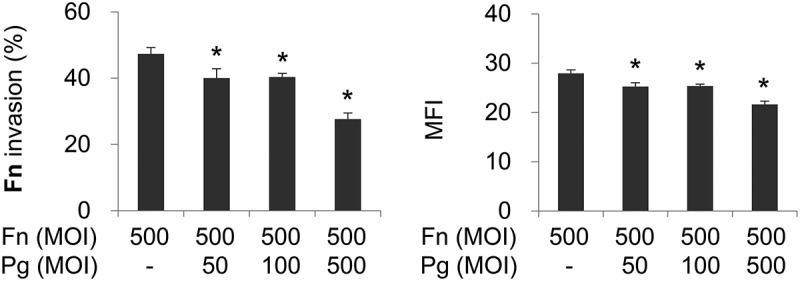
*Porphyromonas gingivalis* infection reduces the invasion of coinfecting *Fusobacterium nucleatum* into gingival epithelial cells. Human Oral Keratinocyte-16B (HOK-16B) cells were infected with carboxyfluorescein diacetate succinimidyl ester (CFSE)-labeled *F. nucleatum* and unlabeled *P. gingivalis* at the indicated multiplicities of infection (MOIs) for 4 h. The cells were analyzed using flow cytometry after quenching the extracellular fluorescence from bacteria attached to the cell surface with trypan blue. The percentage of cells containing *F. nucleatum* (left panel) and the mean fluorescence intensity (MFI; right panel) are shown as the mean ± standard deviation. Representative data from three independent experiments are shown. Fn, *F. nucleatum*; Pg, *P. gingivalis*. *p < 0.05 compared with *F. nucleatum* monoinfection.

To examine whether *P. gingivalis* affects invasion of coinfecting *F. nucleatum*, HOK-16B cells were infected with CFSE-labeled *F. nucleatum* in the presence or absence of *P. gingivalis*. In monoinfection, when analyzed by flow cytometry, *F. nucleatum* invaded into >40% of HOK-16B cells (Fig. 1). This result is consistent with a previous study that reported the high invasive capacity of *F. nucleatum* into gingival epithelial cells [24]. When coinfected with *P. gingivalis*, invasion of *F. nucleatum* into HOK-16B cells significantly decreased in a MOI-dependent manner (Fig. 1). When it was tested whether *P. gingivalis* also inhibited invasion of other oral bacteria, such as *Tannerella forsythia* and *Streptococcus oralis*, invasion of *T. forsythia* was enhanced by *P. gingivalis* coinfection, whereas invasion of *S. oralis* was not affected by the presence of *P. gingivalis* (Supplementary Fig. 1). These results suggest that *P. gingivalis* differentially affects the invasion of co-infecting bacteria and that the suppression of *F. nucleatum* invasion by *P. gingivalis* might result from specific interactions among *F. nucleatum, P. gingivalis*, and the gingival epithelial cells.

### Gingipains of coinfecting *P. gingivalis* are required for the suppression of *F. nucleatum* invasion

**Figure 2.**
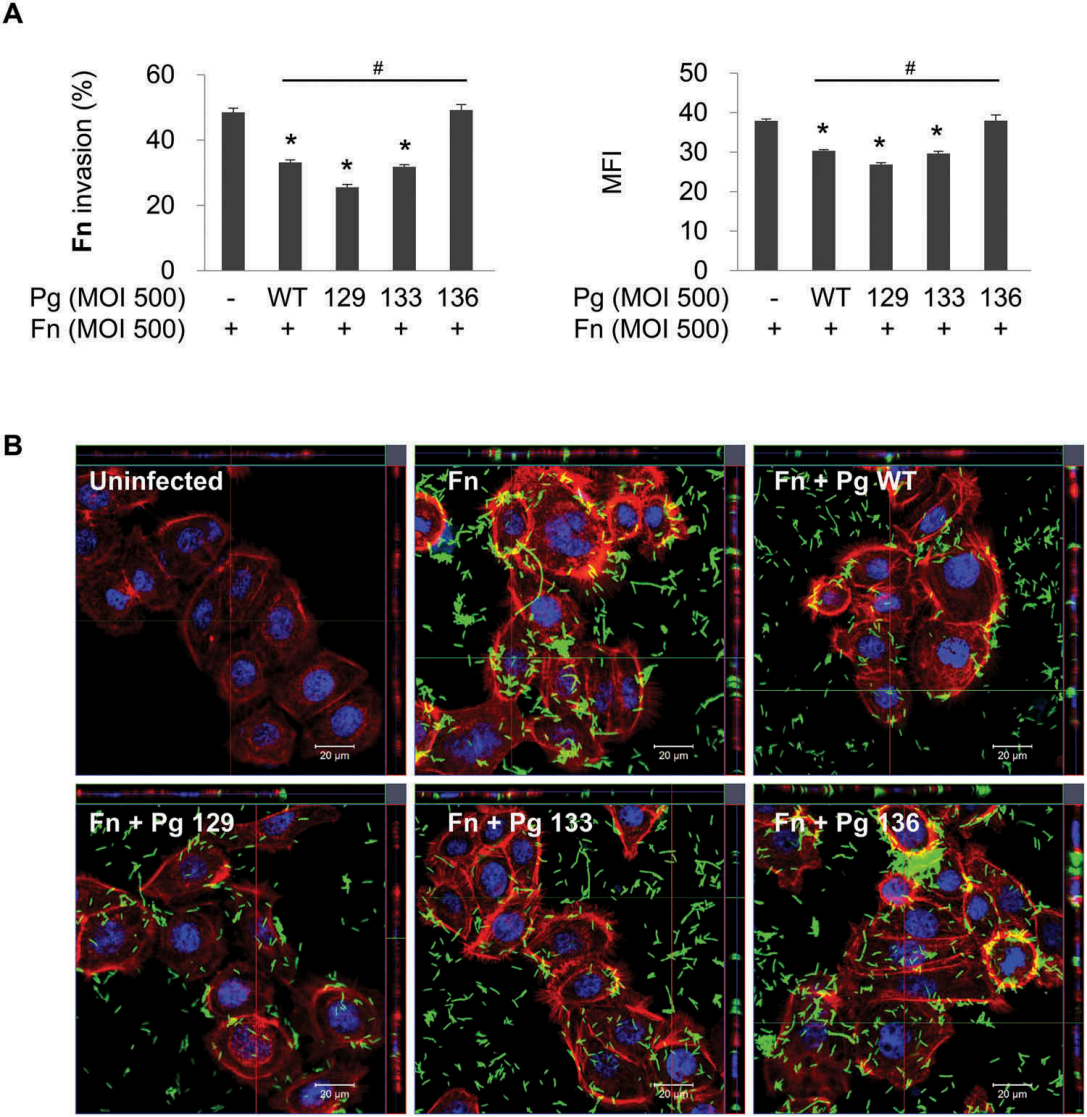
Gingipain deficiency reverses the suppression of *F. nucleatum* invasion by *P. gingivalis*. HOK-16B cells were infected with CFSE-labeled *F. nucleatum* and unlabeled wild-type *P. gingivalis* or gingipain mutants at a MOI of 500 for 4 h. (A) Cells were analyzed using flow cytometry after quenching the extracellular fluorescence from the bacteria attached to the cell surface with trypan blue. The percentage of cells containing *F. nucleatum* (left panel) and the MFI (right panel) are shown as the mean ± standard deviation. Representative data from three independent experiments are shown. (B) The cells were examined under a confocal laser scanning microscope after staining for F-actin (red) and nuclei (blue). CFSE-labeled *F. nucleatum* is displayed in green. Fn, *F. nucleatum*; Pg, *P. gingivalis*; WT, *P. gingivalis* ATCC 33277; 129, KDP129 (*kgp*^−^); 133, KDP133 (*rgpA*^−^ *rgpB*^−^); 136, KDP136 (*kgp*^−^ *rgpA*^−^ *rgpB*^−^). *p < 0.05 compared with *F. nucleatum* monoinfection; ^#^*p* < 0.05 compared with coinfection with wild-type *P. gingivalis.*

To investigate the role of gingipains in the inhibitory effect of *P. gingivalis* on *F. nucleatum* invasion, gingipain mutant strains of *P. gingivalis* were used for coinfection. Kgp- and Rgp-deficient mutants (KDP129 and KDP133, respectively) exhibited inhibitory effects, similar to the wild-type, on *F. nucleatum* invasion. However, the gingipain-null mutant (KDP136) did not decrease the invasion of *F. nucleatum* into HOK-16B cells (Fig. 2(A)). Confocal microscopy also revealed that deletion of all gingipains abrogates the inhibition of *F. nucleatum* invasion by *P. gingivalis* (Fig. 2(B)). These results indicate that the presence of either Kgp or Rgp in *P. gingivalis* exerts an inhibitory action on *F. nucleatum* invasion. In contrast, in HGFs and macrophages, *P. gingivalis* enhanced *F. nucleatum* invasion and phagocytosis, respectively, and gingipains contributed to the enhancement (Supplementary Fig. 2), suggesting that the effect of *P. gingivalis* and gingipains on the internalization of *F. nucleatum* depends on the cell type.

### Inhibition of PI3K attenuates *F. nucleatum* invasion

**Figure 3.**
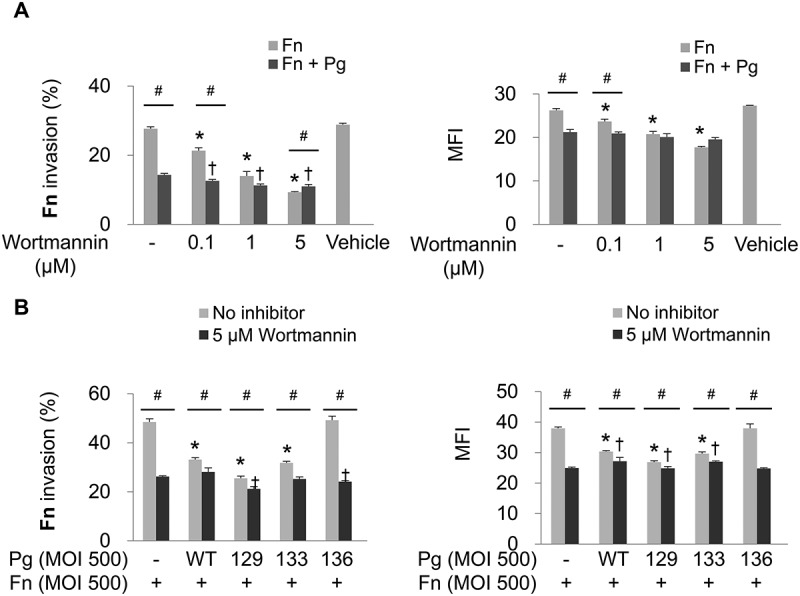
Invasion of *F. nucleatum* is suppressed by PI3K inhibition. (A) HOK-16B cells were preincubated with wortmannin at the indicated concentration for 30 min and then infected with CFSE-labeled *F. nucleatum* and unlabeled wild-type *P. gingivalis* at a MOI of 500 for 4 h. The percentage of cells containing *F. nucleatum* (left panel) and the MFI (right panel) are shown as the mean ± standard deviation. *p < 0.05 compared with cells without preincubation with wortmannin in *F. nucleatum* monoinfection; ^†^*p* < 0.05 compared with cells without preincubation with wortmannin in mixed infection with *F. nucleatum* and *P. gingivalis*; ^#^*p* < 0.05 compared between the two groups. (B) HOK-16B cells were preincubated with 5 μM of wortmannin for 30 min and infected with CFSE-labeled *F. nucleatum* and unlabeled wild-type *P. gingivalis* or gingipain mutants at a MOI of 500 for 4 h. *p < 0.05 compared with *F. nucleatum* monoinfection in the absence of wortmannin; ^†^*p* < 0.05 compared with *F. nucleatum* monoinfection in the presence of wortmannin; ^#^*p* < 0.05 compared between the two groups. Representative data from three independent experiments are shown. Fn, *F. nucleatum*; Pg, *P. gingivalis*; WT, *P. gingivalis* ATCC 33277; 129, KDP129 (*kgp*^−^); 133, KDP133 (*rgpA*^−^ *rgpB*^−^); 136, KDP136 (*kgp*^−^ *rgpA*^−^ *rgpB*^−^).

In addition to the numerous cellular functions of the PI3K/AKT signaling pathway, such as cell survival, proliferation, apoptosis, and protein synthesis [27], the pathway has been reported to be involved in the internalization of some pathogens into host cells [28–31]. To determine whether the PI3K signaling pathway is involved in *F. nucleatum* invasion, HOK-16B cells were infected with *F. nucleatum* in the presence of wortmannin, a commonly used inhibitor of PI3K. In monoinfection, wortmannin significantly inhibited invasion of *F. nucleatum* into HOK-16B cells in a dose-dependent manner, whereas when coinfected with wild-type *P. gingivalis*, the inhibitory effect of wortmannin on *F. nucleatum* invasion was negligible (Fig. 3(A)). Upon coinfection with Kgp- or Rgp-deficient *P. gingivalis* (KDP129 or KDP133, respectively), only a slight decrease in *F. nucleatum* invasion by wortmannin was observed. However, invasion of *F. nucleatum* was significantly decreased during coinfection with the gingipain-null mutant (KDP136) to the same degree as the decrease in the monoinfection resuling from wortmannin treatment (Fig. 3(b)).

### *P. gingivalis* degrades PI3K and AKT, thereby abrogating basal activation of the PI3K/AKT pathway

**Figure 4.**
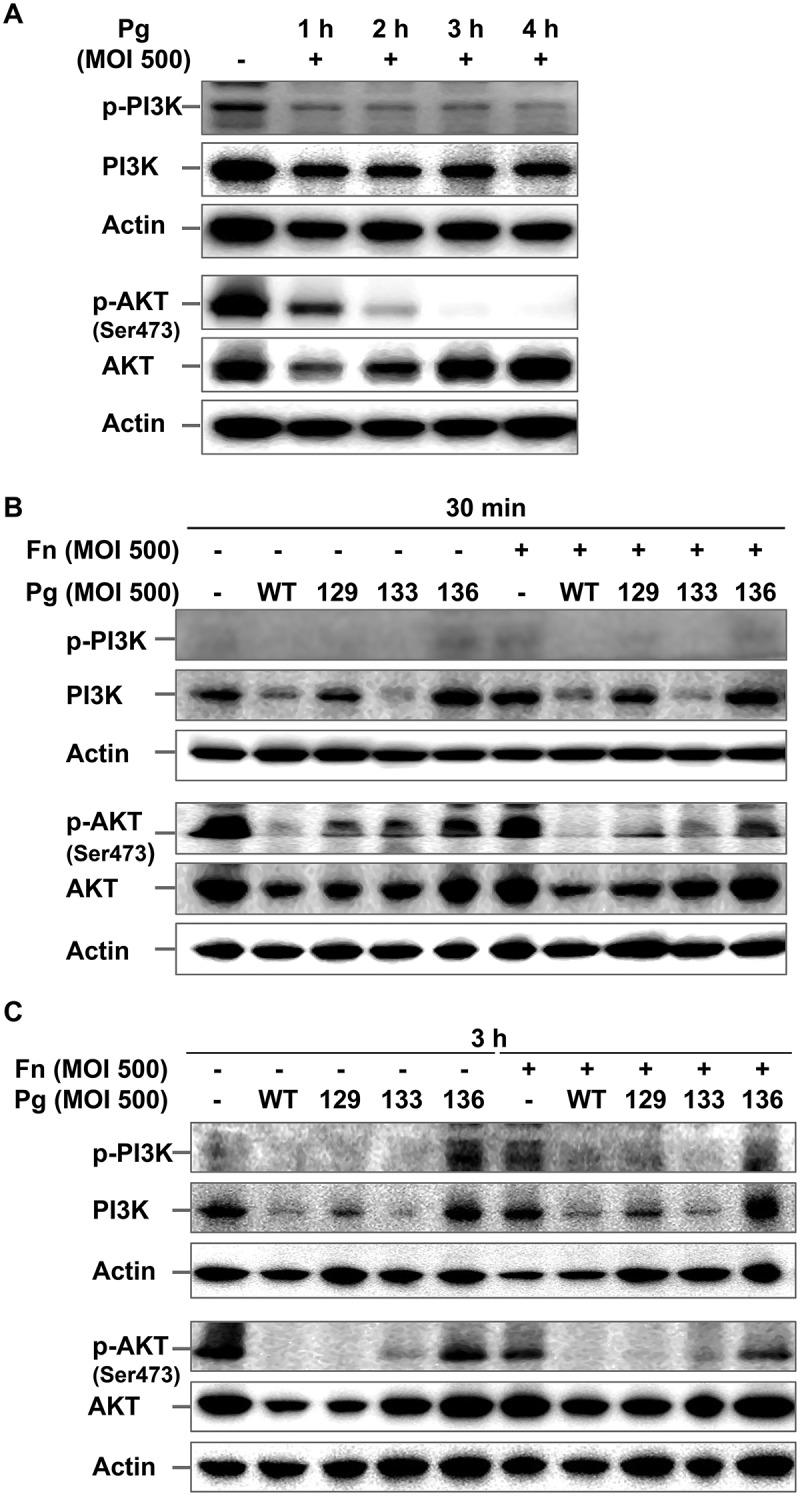
*P*. *gingivalis* inactivates the PI3K/AKT pathway in a gingipain-dependent manner. (A) HOK-16B cells were infected with wild-type *P. gingivalis* at a MOI of 500 for the indicated time. (B) and (C) HOK-16B cells were infected with wild-type *P. gingivalis* or gingipain mutants in the presence or absence of *F. nucleatum* at a MOI of 500 for 30 min (B) or 3 h (C). The levels of phosphorylated and total forms of PI3K and AKT were analyzed by immunoblot analysis. Fn, *F. nucleatum*; Pg, *P. gingivalis*; WT, *P. gingivalis* ATCC 33277; 129, KDP129 (*kgp*^−^); 133, KDP133 (*rgpA*^−^ *rgpB*^−^); 136, KDP136 (*kgp*^−^ *rgpA*^−^ *rgpB*^−^).

Because the inhibitory effect of wortmannin on *F. nucleatum* invasion was minute during coinfection with *P. gingivalis* expressing gingipains, it was hypothesized that *P. gingivalis*, in particular its gingipains, could affect the PI3K signaling pathway. To test this hypothesis, the phosphorylation status of PI3K and AKT in HOK-16B cells was examined after infection with *P. gingivalis*. Infection with wild-type *P. gingivalis* led to a decrement in the level of phosphorylated PI3K, as well as its total form for up to 4 h post infection (Fig. 4(A)). The level of total AKT level also markedly decreased after 1 h of *P. gingivalis* infection and recovered 4 h post infection. However, AKT phosphorylation remained abated for 4 h of *P. gingivalis* infection (Fig. 4(A)). *P*. *gingivalis*–induced decreases in phospho-PI3K, phospho-AKT, total PI3K, and total AKT were observed as early as 30 min after infection with wild-type *P. gingivalis* (Fig. 4(B)). The levels of the phosphorylated and total forms of PI3K and AKT were also reduced after both 30 min and 3 h of infection with Kgp- and Rgp-deficient mutants (KDP129 and KDP133, respectively), but not after infection with the gingipain-null mutant (KDP136; Fig. 4(B) and (C)). Infection with *F. nucleatum* did not affect the phosphorylated or total form of PI3K and AKT during monoinfection, and changes in their levels after coinfection with *F. nucleatum* and *P. gingivalis* were consistent with monoinfection with *P. gingivalis* (Fig. 4(B) and (C)).

### P. gingivalis *with Rgp mutation promoted the intracellular survival of* F. nucleatum

**Figure 5.**
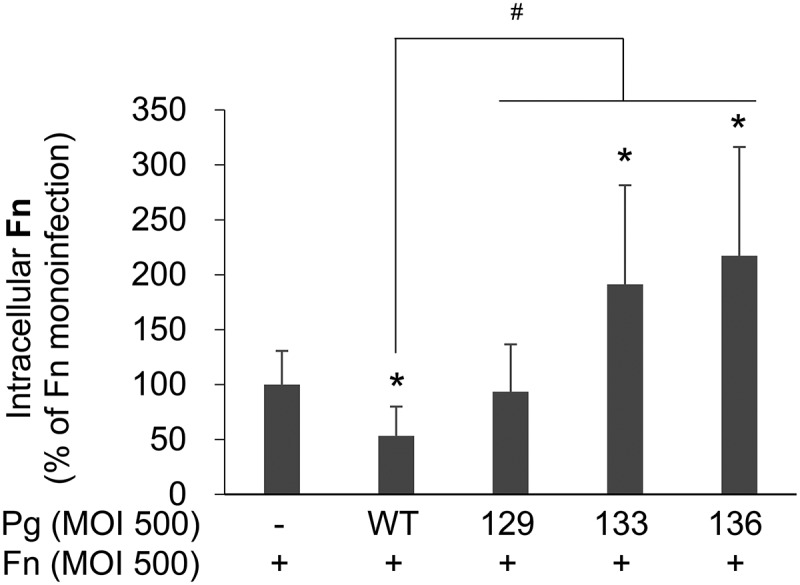
More intracellular *F. nucleatum* remains viable after coinfection with *P. gingivalis* with Rgp mutation than in monoinfection. After HOK-16B cells were infected with *F. nucleatum* in the presence or absence of wild-type *P. gingivalis* or gingipain mutants at a MOI of 500 for 4 h, extracellular bacteria were killed by incubation with antibiotics for 1 h. After incubation in fresh media for 12 h, the cells were lysed, and the lysates were plated on brain heart infusion blood agar plates containing vancomycin. The data represent the mean ± standard deviation of four independent experiments. *p < 0.05 compared with *F. nucleatum* monoinfection; ^#^*p* < 0.05 compared between the two groups. Fn, *F. nucleatum*; Pg, *P. gingivalis*; WT, *P. gingivalis* ATCC 33277; 129, KDP129 (*kgp*^−^); 133, KDP133 (*rgpA*^−^ *rgpB*^−^); 136, KDP136 (*kgp*^−^ *rgpA*^−^ *rgpB*^−^).

Next, the study examined whether intracellular survival of *F. nucleatum* is affected by coinfecting *P. gingivalis*. Twelve hours after the antibiotic protection, the number of intracellular *F. nucleatum* in the monoinfection group remained higher than in the group coinfected with wild-type *P. gingivalis* (Fig. 5). However, after coinfection with Kgp-deficient mutant KDP129, the number of intracellular *F. nucleatum* was similar to that after monoinfection (Fig. 5). Interestingly, after coinfection with Rgp-deficient mutant KDP133 or the gingipain null mutant KDP136, more *F. nucleatum* remained viable within the infected cells than after monoinfection (Fig. 5). Considering that KDP129 and KDP133 suppressed *F. nucleatum* invasion and that during coinfection with KDP 136 *F. nucleatum* invasion was similar to monoinfection, this result suggests that the intracellular survival of *F. nucleatum* is facilitated by coinfecting gingipain mutants of *P. gingivalis*. Few *F. nucleatum* survived after 18 h of infection, regardless of coinfection with *P. gingivalis* (data not shown).

## Discussion

The present study demonstrated that *P. gingivalis* compromised invasion of coinfecting *F. nucleatum* into gingival epithelial cells in a gingipain-dependent manner. While PI3K inhibition reduced *F. nucleatum* invasion into gingival epithelial cells, *P. gingivalis* infection inactivated the PI3K/AKT pathway. *P*. *gingivalis* with Rgp mutation seemed to facilitate the survival of intracellular *F. nucleatum*.

*F*. *nucleatum* invades into gingival epithelial cells via a zipper mechanism [32], and a pre-FadA-mature FadA complex mediates attachment and invasion of *F. nucleatum* into gingival epithelial cells [20]. Infection of gingival epithelial cells by *F. nucleatum* increases the expression of antimicrobial peptides and cytokines, such as human beta defensin (HBD)-2, HBD-3, interleukin (IL)-6, and IL-8 [22,24,32,33]. In particular, the induction of HBD-2, HBD-3, and IL-8 production by *F. nucleatum* depends on bacterial invasion and endosomal maturation [24,34]. After invasion, endosomes containing *F. nucleatum* rapidly fuse with lysosomes, and intracellular *F. nucleatum* is cleared via endolysosomal degradation [24].

Due to the polymicrobial nature of the pathogenesis of periodontitis, it is important to understand the polymicrobial interactions as well as the virulence mechanisms of each periodontopathogen. Polymicrobial infections of periodontal pathogens often result in synergistic pathogenicity [35,36]. Mixed infection with *F. nucleatum* and *P. gingivalis* has been shown to aggravate alveolar bone loss in a mouse model of experimental periodontitis [36] and abscess formation in a murine abscess model [35]. However, the mechanism of synergism between these two bacterial species remains unclear. A recent study demonstrated that coaggregation of *F. nucleatum* with a capsulated *P. gingivalis* strain contributes to the augmented virulence of the mixed infection [37]. Previous studies have shown that *P. gingivalis* inhibits the induction of HBD-2 and IL-8 expression in gingival epithelial cells by *F. nucleatum* [22,38,39], which might be partly explained by the finding in the present study that *P. gingivalis* restricts *F. nucleatum* invasion. A previous study showed that invasion by *P. gingivalis* into gingival epithelial cells is significantly inhibited by preceding infection with *F. nucleatum* [40]. Conversely, it may be possible that *P. gingivalis* invasion into gingival epithelial cells interferes with cellular pathways required for subsequent invasion by *F. nucleatum* into the cells, although in the present study sequential infection with *P. gingivalis* followed by *F. nucleatum* was not tested. In contrast to the present observation, a recent study reported that mixed infection with *P. gingivalis* increased *F. nucleatum* invasion into keratinocytes derived from mouse palatal tissues [37]. The discrepancy might result from differences in the cell types and bacterial strains used in the studies.

Gingipains are essential for the establishment of *P. gingivalis* infection, as well as for immune subversion by *P. gingivalis* [6–10]. In the process of *P. gingivalis* invasion into gingival epithelial cells, gingipains seem to play multiple roles with conflicting effects. A previous study reported that inhibition of *P. gingivalis* proteases results in reduced *P. gingivalis* invasion. Rgp is required for maturation of *P. gingivalis* FimA, a major adhesin for gingival epithelial cells [41]. Additionally, adhesin domains of Kgp and RgpA have been shown to participate in attachment of *P. gingivalis* to oral epithelial cells [42,43]. On the contrary, it also has been reported that Rgp mutation substantially increases the bacterial invasion, suggesting that Rgp may modulate the bacterial attachment by cleaving cell surface receptors [42,44].

The role of gingipains in the interaction between *P. gingivalis* and *F. nucleatum* has not been well studied yet. In multispecies biofilms, a deficiency of gingipains in *P. gingivalis* does not affect the growth of *F. nucleatum* [45]. A previous study showed that *P. gingivalis* gingipains attenuate the *F. nucleatum*–induced IL-8 response by degrading IL-8 [38]. In the present study, while gingipains contributed to the enhancement of the internalization of *F. nucleatum* into gingival fibroblasts and macrophages, they were responsible for the inhibition of *F. nucleatum* invasion into gingival epithelial cells by *P. gingivalis*. These results suggest that *P. gingivalis* gingipains differentially affect interactions between *F. nucleatum* and host cells depending on cell types. While receptors for *F. nucleatum* in gingival fibroblasts and macrophages remain unknown, it has been demonstrated that E-cadherin in colon epithelial cells and VE-cadherin in endothelial cells are required for FadA-mediated intracellular invasion by *F. nucleatum* [16,46]. Because gingival epithelial cells, including HOK-16B cells, express E-cadherin [47–49], it is likely that FadA-dependent invasion by *F. nucleatum* into gingival epithelial cells is also mediated by E-cadherin. Inhibition of *F. nucleatum* invasion into gingival epithelial cells by *P. gingivalis* might be attributable to degradation of E-cadherin by *P. gingivalis* gingipains, which has been previously reported [48]. However, further research is required to explore this possibility. Intracellular survival of *F. nucleatum* seemed to be increased by *P. gingivalis* with Rgp mutation but not by wild-type *P. gingivalis*. Because colocalization of intracellular *F. nucleatum* with lysosomes was not affected by *P. gingivalis* coinfection (data not shown), further studies are needed to elucidate how Rgp mutation affects the intracellular fate of coinfecting *F. nucleatum*.

The PI3K/AKT pathway has been reported to be exploited by a number of pathogens, such as group A streptococcus, group B streptococcus, and *T. forsythia*, for their internalization into host cells [28–30]. Conversely, it has also been reported that the pathway restricts the establishment of certain bacteria, such as Dr + *Escherichia coli* [31]. The present result demonstrating a decrease in *F. nucleatum* invasion caused by wortmannin, a PI3K inhibitor, indicates that invasion of *F. nucleatum* into gingival epithelial cells also requires the PI3K pathway. In addition, gingipain-expressing *P. gingivalis* inactivated the PI3K/AKT pathway, a result that is consistent with a previous study [50], and the inhibitory effect of the PI3K inhibitor on *F. nucleatum* invasion was minute in coinfection with gingipain-expressing *P. gingivalis*. These findings imply that the suppressive effect of *P. gingivalis* on *F. nucleatum* invasion is partially mediated by attenuation of the PI3K/AKT pathway by gingipains. Additional studies will be required to investigate whether the effects of gingipains on other cellular receptors, intracellular pathway, or adhesins of *F. nucleatum* are involved.

In summary, *P. gingivalis* suppresses the invasion of coinfecting *F. nucleatum* into gingival epithelial cells, to which inactivation of the PI3K/AKT pathway by its gingipains might partly contribute. Because *P. gingivalis* degrades intercellular junction complexes in the epithelium [49], *P. gingivalis* may guide *F. nucleatum* toward a paracellular pathway in order to avoid intracellular degradation. Furthermore, by inhibiting and degrading antimicrobial peptides and chemokines [22,38,39], *P. gingivalis* provides a local environment favorable for the survival of *F. nucleatum*, which might allow *F. nucleatum* to invade into deeper periodontal tissues. Further research on this possibility might help us understand how *P. gingivalis* acts as a keystone pathogen.

## Supplementary Material

Supplementary_materials.zip
