# Supplementary figures and images for: Porphyromonas gingivalis suppresses invasion of Fusobacterium nucleatum into gingival epithelial cells
Source: J Oral Microbiol. 2017 Jun 12;9(1):1320193. doi: 10.1080/20002297.2017.1320193 (PMC5508355; doi:10.1080/20002297.2017.1320193)

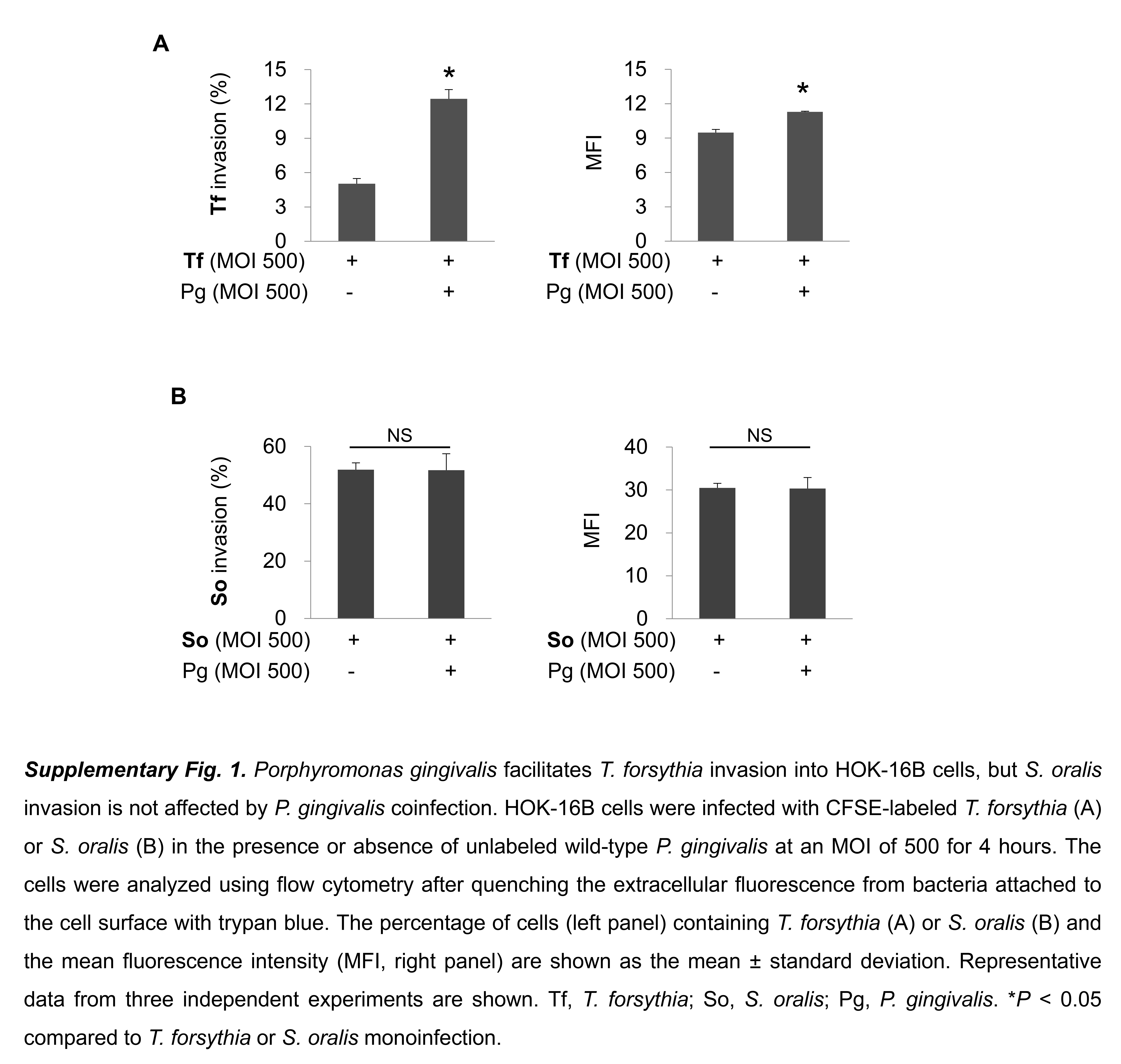

Supplement: Supplementary_materials.zip [file zjom_a_1320193_sm5149.zip › Supplementary materials/Supplementary Fig 1.tif]
